# Computational Analysis of Correlations between Image Aesthetic and Image Naturalness in the Relation with Image Quality

**DOI:** 10.3390/jimaging8060166

**Published:** 2022-06-09

**Authors:** Quyet-Tien Le, Patricia Ladret, Huu-Tuan Nguyen, Alice Caplier

**Affiliations:** 1 Faculty of Information Technology, Vietnam Maritime University, 484 Lach Tray, Le Chan, Hai Phong 04000, Vietnam; huu-tuan.nguyen@vimaru.edu.vn; 2 GIPSA Lab, Grenoble Alpes University, 11 Rue des Mathematiques, Grenoble Campus BP 46, CEDEX, F-38402 Saint Martin d’Heres, France; patricia.ladret@gipsa-lab.grenoble-inp.fr (P.L.); alice.caplier@gipsa-lab.grenoble-inp.fr (A.C.)

**Keywords:** image quality, image aesthetic, image naturalness, human visual perception, image quality assessment, image aesthetic assessment, image naturalness assessment, quality of experience

## Abstract

The main purpose of this paper is the study of the correlations between Image Aesthetic (IA) and Image Naturalness (IN) and the analysis of the influence of IA and IN on Image Quality (IQ) in different contexts. The first contribution is a study about the potential relationships between IA and IN. For that study, two sub-questions are considered. The first one is to validate the idea that IA and IN are not correlated to each other. The second one is about the influence of IA and IN features on Image Naturalness Assessment (INA) and Image Aesthetic Assessment (IAA), respectively. Secondly, it is obvious that IQ is related to IA and IN, but the exact influence of IA and IN on IQ has not been evaluated. Besides that, the context impact on those influences has not been clarified, so the second contribution is to investigate the influence of IA and IN on IQ in different contexts. The results obtained from rigorous experiments prove that although there are moderate and weak correlations between IA and IN, they are still two different components of IQ. It also appears that viewers’ IQ perception is affected by some contextual factors, and the influence of IA and IN on IQ depends on the considered context.

## 1. Introduction

**Figure 1:**
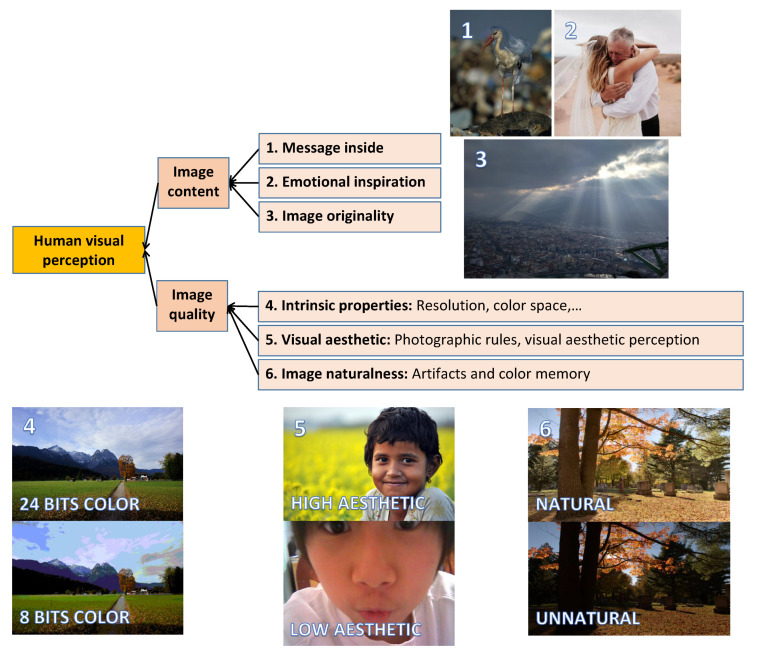
Overview of image aspects having an influence on human visual perception.

In recent years, there have been more and more personal devices integrated with digital cameras, such as smartphones, tablets, and laptops. It has led to a dramatic increase in the number of photos taken day by day, so users’ storage tends to be filled very fast. Therefore, evaluating photos to keep the best ones and remove the worst ones becomes an essential need. This task is traditionally performed based on the human visual system. Figure 1 shows an overview of image factors affecting human visual perception. Those factors are categorized into two groups: image content and Image Quality (IQ). On one side, image content obviously has a great influence on human visual perception. This group contains three factors: “message inside”, “emotional inspiration” and “image originality”. In Figure 1(1), the first photo is an example of “message inside” with a bird stuck in a plastic bag. Although the content looks simple, it contains a hidden message related to the environment, such as “let’s save animals”, “stop consuming plastic bags” or “our planet is destroyed”. Regarding Figure 1(2), some people might not have any special feelings about the photo, but the hug between the bride and her father could remind other people of their family members or a personal memory. The value of the photo is “emotional inspiration”. In Figure 1(3), a rare moment of a cloudy sky with light rays makes the photo different from other photos of the same landscape. Although there is no hidden message or emotional inspiration in this case, the originality makes the photo special.

**Figure 2:**
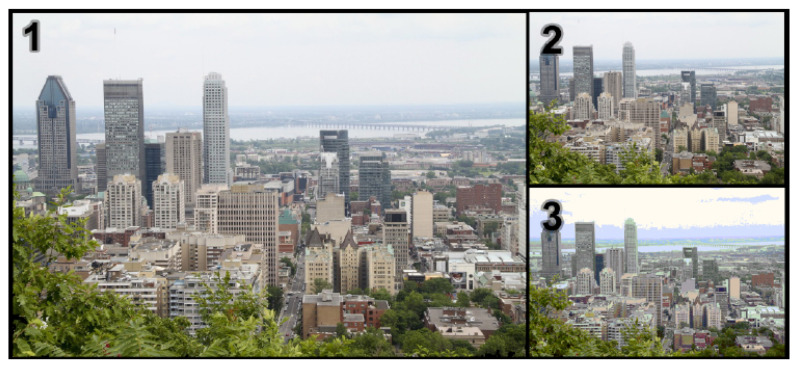
Examples of intrinsic properties. The first photo has a higher resolution than the others, while the color depth of the third photo is shallower than those of the two first ones.

On the other hand, image perception might be based on Image Quality (IQ), which is not supposed to be related to image content. In this paper, we are focusing on IQ only. IQ, in this study, is defined in an explicit way. It is generally based on three notions: intrinsic properties, Image Aesthetic (IA—this work considers this notion related to visual aesthetic only, other aspects related to image content are not considered in this study) and Image Naturalness (IN). “Intrinsic properties” is a notion related to some technical aspects of photos, such as resolution, color space, color depth and image format (see Figure 1(4) and Figure 2). This notion mostly refers to the properties of the image acquisition device, and it is not related to any external factors induced by viewers’ experiences or contexts. In the past, intrinsic properties were the main factor influencing IQ since acquisition device performances were not so good (low resolution, optical or chromatic aberrations). However, this has been reduced because of the improvement in the technology, so the role of intrinsic properties in IQ is less and less significant. That is the reason why, in our study, we make the assumption that intrinsic properties do not influence IQ on the image databases we are dealing with.

**Figure 3:**
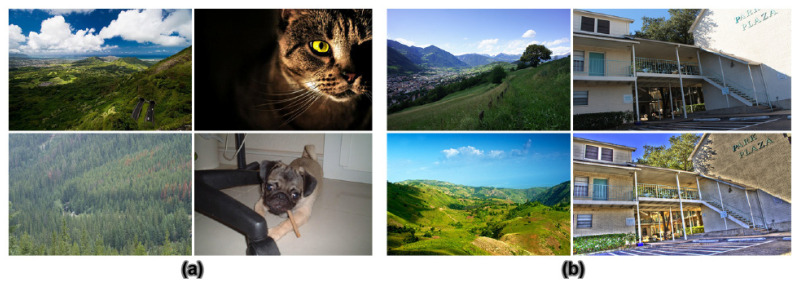
(**a**) IA illustrations: examples of high aesthetic images are in the first row, while the second row contains examples of low aesthetic images. (**b**) IN illustrations: examples of natural images are in the first row, while the second row contains examples of unnatural images.

On the contrary, IA is the measure of how aesthetically a photo fulfills the observer’s expectation based on photography rules and individual visual aesthetic perception (see Figure 1(5) and Figure 3a). This notion is related to what happens in a viewers’ mind when they look at a photo.

On the side of IN, this notion is both related to artifacts induced by some image processing algorithms and to an individual feeling [1] (see Figure 1(6) and Figure 3b). Regarding artifacts, IN is affected by strong visible clues detected by viewers’ eyes, so the unnaturalness feeling comes from annoying artifacts induced by camera sensors, image processing algorithms (compressing, tone-mapping), image format or file transfer (see bottom right photo in Figure 3b). In contrast, the feeling of naturalness and unnaturalness might also come from the viewer’s experience and memory (see bottom left photo in Figure 3b). When viewing a photo, observers compare the scene in the photo to the reality retrieved from their memory (what they have seen) to find differences and similarities, so the feelings are not the same for all viewers. There is also a part of subjectivity in IN perception.

To sum up, this paper focuses on subjective aspects of IQ, including IA and IN. Intrinsic properties are not supposed to influence IQ in our study. Although there are many studies about IQ, IA and IN, the potential relationships between IA, IN and IQ are still an open question. The main purpose of this study is to clarify the correlations between IA and IN—two aspects of IQ—and to investigate the relationships between contextual factors and the impact of IA and IN on IQ. Understanding those correlations could be a potential base to simulate human visual perception and enhance IQ based on IA and IN. A ground truth with both IA and IN data from subjective experiments does not exist, although many studies of IA and IN have been performed over decades. Therefore, instead of using subjective data, this work is approached computationally. IA and IN perception is modeled based on a subjective ground truth of IA and IN separately. By using those simulated models, IA and IN might be measured. The simulated data are then used to analyze the correlations between IA and IN in relation to IQ.

## 2. State of the Art

### 2.1. Image Quality

IQ can be approached from different angles. One common way is to implicitly define IQ with regard to a given Image Quality Assessment (IQA) protocol: either Full Reference Image Quality Assessment (FRIQA), No Reference Image Quality Assessment (NRIQA) or reduced reference IQA. On the side of FRIQA, there are many purposes for image transformation, such as image compression, tone-mapping, steganography and image enhancement. It is assumed that the chain of operations induces negative effects on the quality of the transformed versions compared to the quality of the original version. Therefore, IQ depends on the negative effects induced on the original photo. As a consequence, IQ could be estimated as the similarity between the transformed image and the original image. In other words, IQ is the measurement of how close the transformed versions and the original ones are. In the former, the simplest metric based on signal processing theory is to compute the differences of corresponding pixels between versions. Classical IQA methods are based on distortions computed on pixel values, such as mean squared error, root mean squared error and peak signal to noise ratio. Although it is a simple approach, the obtained results are not really suitable for IQA because human perception is more complicated than the way machines process signals. The computed difference does not always match with visual perception. A more efficient approach is based on human visual system properties. The approach is based on psychological and physical characteristics of the human visual system to compute the visual quality of photos, such as luminance, contrast, structure, fidelity criterion and similarity indexes [2,3,4,5,6,7,8].

IQ is not always estimated through the similarities between the transformed version and the original version because original photos without any modifications are not always the best versions. Although original images are not affected negatively by processing methods, artifacts and distortions could still exist in those photos because of many factors, such as camera sensors, camera settings, brightness conditions and motion. In the cases of image restoration and image enhancement processes, the IQ of some transformed versions could be better than that of the original one, so IQ, in this case, is based on the enhancement of bad visibility [9]. IQ is also based on viewers’ preferences [10]. In some cases, an appealing version could be preferred to a version that is more similar to the reference than the appealing one. It appears that the most preferred version is not necessarily the closest to the reference image.

On the side of NRIQA, IQ is, on the contrary, based on viewers’ background, including preference, visual aesthetic perception, color memory and naturalness perception. A lot of metrics have been presented and validated on results coming from subjective experiments to estimate IQ in this context [11,12,13,14,15,16,17,18,19,20,21,22,23]. Ke et al. [11] define IQ based on abstract aesthetic aspects such as composition, color and lighting to classify professional and snapshot photos. Additionally, they consider simplicity, realism and photography technique as the three main factors producing a high-quality image. Similarly, in [12,14,16], IQ is defined from the perspective of visual aesthetics. According to photography rules, Tang et al. [16] propose an NRIQA metric based on professionals’ views, including composition, lighting, color arrangement, camera settings and topic emphasis. In another approach, Hosu et al. [21] define IQ as a technical concept related to some types of degradations, such as over-saturation, noise, aliasing, motion blur, wrong exposure, over-sharpening, color fringing and JPEG artifacts. Using the same approach, IQ is based on visual distortions induced by technical causes (noise, blur, JPEG compression) in [15]. Besides that, the IQ of tone-mapped images are defined in a different way. Yaacoub et al. [18] consider tone-mapped IQ as the balance between luminance contrast and naturalness. In [17], it is assumed that a high-quality tone-mapped image maintains global information, details and naturalness, so IQ, in this case, is described as the combination of luminance, structure and naturalness. In another study, Jiang et al. [19] assume that the IQ of tone-mapped images could be affected because of under exposure, over exposure and losses in Image Naturalness (IN) and Image Aesthetic (IA). They described IQ by using three factors, including brightness in the brightest and darkest regions, IN and IA. Using a different approach, Leisti et al. [13] define IQ based on low-level attributes related to physical aspects (sharpness, lightness, graininess) and high-level attributes related to abstract aspects (individual feelings, viewers’ experiences, naturalness). In [23], Varga et al. pointed out that first digit distributions based on metrics of high-quality images extracted from multiple domains match well with Benford’s law. That study demonstrated that first digit distribution are quality-aware features, and it is possible to achieve high performance in NRIQA with those features. Based on a different approach, multiple global average pooling architectures were used for IQA in [22]. Specifically, a deep model containing multiple inception blocks was attached to average global pooling layers to extract features. Instead of taking patches from the input image, the whole image was passed through a pre-trained model so the proposed architecture could work with images of various resolutions.

Another IQA protocol mentioned here is reduced reference IQA. Only partial information is provided about the reference image. Characteristics or features such as histogram, color saliency map and sharpness map are extracted. In reduced reference IQA, reduced references are somehow like human memory. A person sometimes feels that he/she has seen the scene of a photo but without remembering all of the details. Several IQA metrics based on reduced reference have been introduced: in [24], the IQA metric is based on a divisive normalization image representation coming from a Gaussian scale mixture statistical model of image wavelet coefficients; in [25], the IQA metrics are based on a linear relationship between full reference and reduced reference structural similarity index measures; in [26], the IQA metric is based on an orientation selectivity mechanism for visual content extraction; in [27], the IQA method is based on saliency maps and texture similarity between high-resolution and low-resolution photos.

Generally, in either FRIQA or other IQA protocols, IQ refers to the measurement of how photos satisfy viewers. The satisfaction of viewers mainly depends on visual aesthetic perception and the feeling of how close photos and real scenes are. Therefore, it could be seen that the two factors IA and IN play important roles in IQ.

### 2.2. Image Aesthetic

The questions of how a photo is captured as well as how a viewer enjoys and criticizes the photo leads to the visual perception of aesthetics in photography, so a part of IA perception is based on photographic rules. Many studies have been made to model IA. In [28], IA is described based on rules of composition, depth of field, salient object, opposing colors and natural illumination. In another study, Marchesottian et al. [29] introduced a description of IA using a bag of visual word descriptors, Fisher vector and GIST descriptors. Besides that, in [30], IA is evaluated based on the combination of simplicity, patterns of harmony and rhythm, colorfulness, composition and sharpness. In a similar approach, Aydin et al. [31] introduce an IA signature concept based on sharpness, clarity, tone, depth and colorfulness features. However, not all aesthetic aspects are describable, so it does not mean that following photography rules always produces a high aesthetic photo, and on the contrary, a beautiful photo might not follow those rules. As a consequence, there is a part of subjectivity in IA perception. A deep learning approach might be a good solution to describe the subjective aspects. Various studies on IA using deep learning have been proposed, such as the Image Aesthetic Assessment (IAA) model based on the combination of a retrieval system and a deep Convolutional Neural Network (CNN) presented in [32], the double-column deep CNN architecture using two parallel CNNs based on global and local features proposed in [33], a CNN including 3 kinds of layers: transferred layers, scene convolutional layers and fully connected layers, evaluating the IA of multi-scenes in [34], the IAA model based on the deep learning technique, image classification and image segmentation introduced in [35]. Moreover, Hii et al. [20] proposed a deep model exploiting multiple inception blocks pooled by global average pooling layers. They also integrated textual information with visual information to perform IQA. The experimental results in that work demonstrated a good performance of the proposed architecture.

### 2.3. Image Naturalness

Different definitions of IN have been introduced. In [36,37,38], IN is described as the degree of correspondence between a photo displayed on a device and the memory of the real-life scene. In [39], Cadik et al. introduce IN as the degree of correspondence between a scene (seen directly) and the corresponding scene in photos based on some technical criteria: brightness, contrast, color reproduction, reproduction of details, simulation of glare, visual acuity and artifacts. In another study, Jiang et al. [19] define IN based on the differences between photos taken with normal exposure and abnormal exposure, in which unnatural photos are described as over or under exposed photos and natural photos are considered as photos captured with normal exposure. In some studies, IN features have been employed for IQA [17,18,19]. Moreover, in [1], IN is based on artifacts induced by some image processing methods (such as halos, blur, lost details) and on the individual feeling (memory, opinion, background).

It could be seen that many efforts to simulate human visual perception have been made. Machines have been trained to understand and measure IQ like humans. In order to understand more about IQ, IA and IN to simulate human visual perception, the correlations between the two aspects of IQ: IA and IN, are studied in this work. Obviously, IA and IN have been described in various computational ways in previous studies as presented above, so a computational approach might be an acceptable choice to study IA and IN.

## 3. Potential Relationships between IA and IN

In order to answer the main purpose of this section, three sub-tasks are considered. The first one is to estimate the correlation between IA and IN features. Secondly, the influence of IA and IN features on Image Naturalness Assessment (INA) and IAA, respectively, is measured. The last task is about the equivalence between high aesthetics and naturalness on the one side and between low aesthetics and unnaturalness on the other side.

Although many IA and IN datasets have been introduced over decades, a dataset with both IA and IN ground truths from subjective experiments does not exist. Therefore, instead of using a dataset with both subjective IA and IN ground truths to evaluate the relationships between IA and IN, an IA dataset and an IN dataset with subjective ground truths are used, and the IN of the IA dataset and the IA of the IN dataset are computed by an INA model and an IAA model, respectively.

In this research, an IA dataset coming from [35] and an IN dataset coming from [1] are considered. On the one hand, the IA dataset contains 1200 high aesthetic images and 1200 low aesthetic images coming from the CUHKPQ dataset [16]. Each photo of the CUHKPQ dataset is evaluated by ten observers, and a photo is considered as “high aesthetic” if at least eight of the ten viewers consider its aesthetic as “high” [16]. Similarly, a photo is labeled as “low aesthetic” if at least eight of the ten viewers consider its aesthetic as “low”. On the other hand, the IN dataset contains 355 natural photos and 515 unnatural photos, each assessed by nine observers. A photo is labeled as “natural” if at least eight of the nine viewers consider it as “natural” and, similarly, a photo is labeled as “unnatural” if it is assessed as “unnatural” by at least eight of the nine observers [1].

Besides that, an IAA model based on the IA feature set learned from [35], and an INA model based on the IN feature set coming from [1] are considered in this paper because of their good performances. First, the IA feature set contains 1024 global features (features learned from the whole image) learned by a deep CNN having a typical architecture with an input layer, an output layer and five convolutional blocks. Specifically, two convolutional layers and a pooling layer are placed in each block. There are 64 × 2, 128 × 2, 256 × 2, 512 × 2, 1024 × 2 kernels in the five blocks, respectively (two layers in each block). The last layer contains two outputs corresponding to the two categories: high visual aesthetic image and low visual aesthetic image. The model is trained on over 18,000 high and low visual aesthetic photos coming from the CUHKPQ dataset [16], and the obtained accuracy is quite impressive at 0.914. Secondly, the IN features are studied in [1]. Various models, including Xception [40], NASNet [41], MobileNet [42], InceptionNet [43], VGG16 [44] and ResNet [45], pre-trained on over 14 million images of the ImageNet dataset for the task of image classification, were considered in that work. The feature selection process described in [46] is applied to select the most relevant features to the task of INA from the features of each pre-trained model. Those models are then transferred to the new purpose of INA by replacing the last layers (all the fully connected layers) of those models and re-training those layers on the considered IN dataset for the INA task. After training and testing the models using the nine reduced feature sets, the highest overall accuracy (0.865) and the best loss (0.139) are obtained with the model using the features learned from the ResNet extractor. In this case, there are no re-trained ResNet layers. The model without the last layer (the fully connected layer) is considered the feature extractor for the proposed model. Specifically, 425 learned features are selected from the 2048 ResNet features by applying the Relief-based feature reduction algorithm [46]. The overall accuracy of the classification is quite good at 0.865.

### 3.1. IA and IN Feature Correlation Analysis

In this work, two common correlation coefficients are employed to measure the correlation between IA and IN features. The first one is the Pearson correlation coefficient [47]. It is a measure of linear correlation between two sets of data. It is computed as the ratio between the covariance of two variables and the product of their standard deviations. In other words, the Pearson correlation draws a line of best fit through the data of two variables and calculates the effect of change in one variable when the other variable changes. For example, the positive correlation between a child’s age and height (in most cases, a child’s height will keep increasing as his/her age increases), and the negative correlation between a vehicle’s speed and traveling time (if a vehicle increases its speed, the time it takes to move decreases, and vice versa). The second coefficient is the Spearman rank correlation [48]. The Spearman rank correlation between two variables is equal to the Pearson correlation between the rank values of those two variables. Both coefficients range from −1 to 1, in which values near 1 and −1 refer to a perfect correlation in which 1 means that if the value of one variable increases, the value of the other variable increases too, while −1 means that if the value of one variable increases, the value of the other variable decreases; 0 reflects no correlation. For the sake of simplicity, the correlation absolute values only are considered. They range from 0 to 1, and the higher absolute value refers to higher correlation.

**Figure 4:**
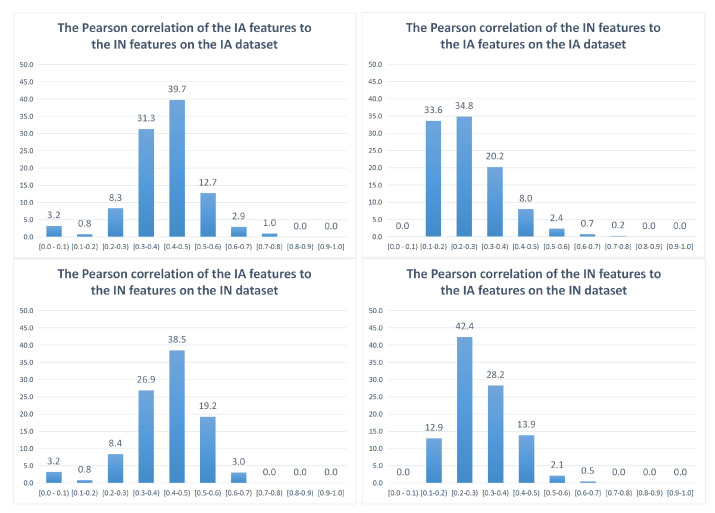
Pearson correlation between IA features and IN features.

**Figure 5:**
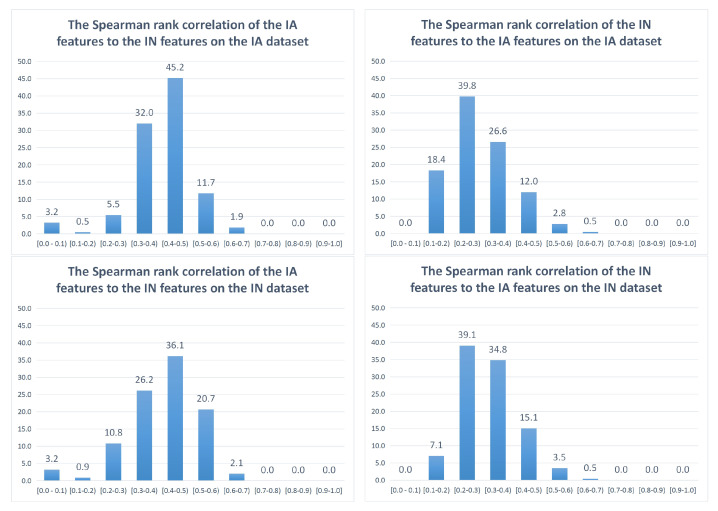
Spearman correlation between IA features and IN features.

To measure the correlation between the IA feature set and the IN feature set, each feature of the two feature sets is computed on *n* images to form an *n-dimensional* vector. The correlation cor[i,j] between the *i*-th IA feature and the *j*-th IN feature is computed as the absolute value of the correlation between the two corresponding *n-dimensional* vectors. The most correlated IN feature to the *i*-th IA feature is determined as the IN feature having the highest correlation ($maxCor_{i}^{IA}$) to the IA feature as in (1), in which 425 is the number of IN features. Similarly, the most correlated IA feature to the *j*-th IN feature is determined as the IA feature having the highest correlation ($maxCor_{j}^{IN}$) to the IN feature as in (2), in which 1024 is the number of IA features. Histograms with ten bins are built based on the highest correlation of each feature computed in the IA dataset [35] and the IN dataset [1], as in Figure 4 and Figure 5.

(1) $$maxCor_{i}^{IA}=\max_{j=1}^{425}\left(cor\left[i,j\right]\right)$$

(2) $$maxCor_{j}^{IN}=\max_{i=1}^{1024}\left(cor\left[i,j\right]\right)$$

Figure 4 shows the Pearson correlation between IA and IN features. Specifically, the horizontal axis represents groups of correlation values, while the vertical axis shows the proportion of features (in percentages). In the IA dataset, it appears that a small part of IA features is highly correlated (the highest correlation of the features is higher than 0.5) to the IN features (15.6%), while the majority of IA features are moderately correlated (the highest correlation of the features ranges from 0.3 to 0.5) to the IN features (71%). Moreover, a minority of IA features (13.4%) is weakly correlated (the highest correlation of the features is lower than 0.3) to the IN features. There is a similar trend in the IN dataset, where 22.2%, 65.4% and 12.4% of IA features are highly correlated, moderately correlated and weakly correlated to the IN features, respectively. In contrast, the IN features seem to be less correlated to the IA features since the majority of IN features are weakly correlated to the IA features (68.4% in the IA dataset and 55.3% in the IN dataset), while only 3.3% and 2.6% of IN features are highly correlated to the IA features in the IA and IN datasets, respectively.

Similarly, Figure 5 shows the Spearman rank correlation between IA features and IN features. The results based on the Spearman rank correlation coefficient are quite similar to the results with the Pearson correlation coefficient. The majority of IA features are highly correlated and moderately correlated to the IN features (13.6% and 77.2% in the IA dataset and 22.8% and 62.3% in the IN dataset, respectively), while a significant part of IN features is weakly correlated to the IA features (58.2% and 46.2% in the IA dataset and the IN dataset, respectively). Besides this, only 3.3% and 4% of IN features are highly correlated to the IA features in the IA dataset and the IN dataset, respectively.

Generally, although there are a few differences between the results based on the Pearson correlation and based on the Spearman rank correlation, both results refer to the same general conclusion that IA features have quite a significant correlation with IN features, but the correlation between IN features and IA features is much weaker.

### 3.2. Are IN and IA Independent or Dependent?

Although the correlation between IA and IN features has been estimated in the previous section, the meaning of the correlation between IA and IN has not been clarified. The considered IA and IN features are learned automatically by deep CNNs [1,35], so they are abstract and not easy to understand. In this subsection, the idea is to check if IN and IA are independent so that they describe two different aspects of IQ or not. To do so, we are going to study if there is an overlap between IA features and IN features first, and then the influence of IN features on IAA and the influence of IA features on INA are going to be evaluated.

#### 3.2.1. Influence of IN Features on IAA

According to the results of Section 3.1, the correlation of IN features with IA features is low. Based on the Pearson coefficient, only 14 IN features (3.3%) are highly correlated to IA features, while the number of weakly correlated IN features is 291 (68.4%). Besides that, there are 120 IN features moderately correlated to IA features (28.2%). It appears that a majority of IN features do not overlap with IA features but the number of moderately correlated IN features is significant (28.2%).

**Figure 6:**
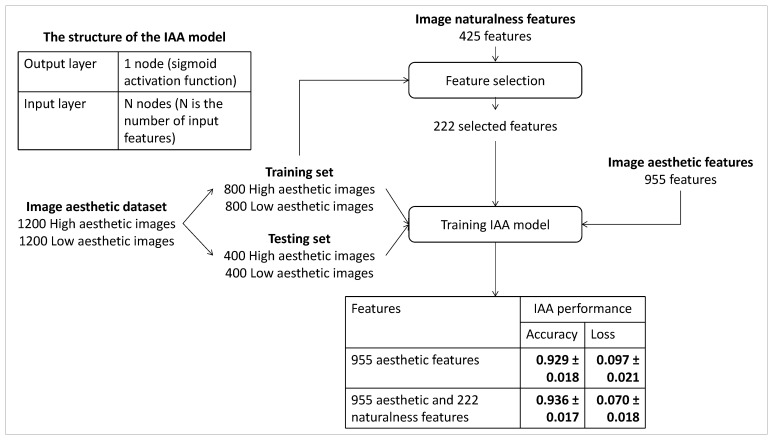
Experiment process of evaluating how IN features improve the performance of IAA.

In order to evaluate the influence of IN features on IAA, we propose to train an IAA model by considering IN features only, including 14 highly correlated, 120 moderately correlated and 291 weakly correlated features. The performance of this model is compared with the one based on IA features only and the one based on the combination of IA and IN features. Figure 6 presents the process of the experiment and the results. The proposed IAA model contains an input layer (the number of input nodes is the number of input features) and an output layer (one output node with sigmoid activation function, a very simple linear model is considered because, in this study, we want to focus on the impact of features instead of the architecture of the model) is trained to perform a binary classification between high and low aesthetic photos. The Adam optimizer and a binary cross-entropy loss function are used, and the batch size is assigned to 100. The learning rate and the number of iterations are set to $5\times 10^{-4}$ and 150, respectively. The IAA model is trained and tested on the IA dataset coming from [35]. The IA dataset labeled by humans is split into a training set containing 1600 images (two0thirds of the dataset) and a testing set including 800 images (one-third of the dataset).

Looking at the results in Figure 6, although the performance of the IAA based on IN features is lower than the one based on IA features (0.915±0.019 versus 0.930±0.018), it is quite impressive. The moderately correlated IN features could be the reason for the good performance with IN features since the IAA based on them has a good performance (0.911±0.020). Moreover, with the number of highly correlated features being small (14 features), the performance of the IAA based on them is bad (0.769±0.029). The results with the weakly correlated IN features are not very impressive since the IAA based on them has a lower performance at 0.874±0.023. Although those features are not overlapping with IA features, they are not related to the IAA task. It could explain the slight increase in accuracy from 0.930 ± 0.018 to 0.946 ± 0.016 when considering the IAA based on the combination of IA features and IN features.

#### 3.2.2. Influence of IA Features on INA

Section 3.1 shows a significant correlation between IA features and IN features. Based on the Pearson coefficient, 228 (22.2%) and 669 (65.4%) IA features are, respectively, highly correlated and moderately correlated to IN features, while the number of weakly correlated IA features is 127 (12.4%). It appears that there is a significant part of IA features that overlap with IN features.

**Figure 7:**
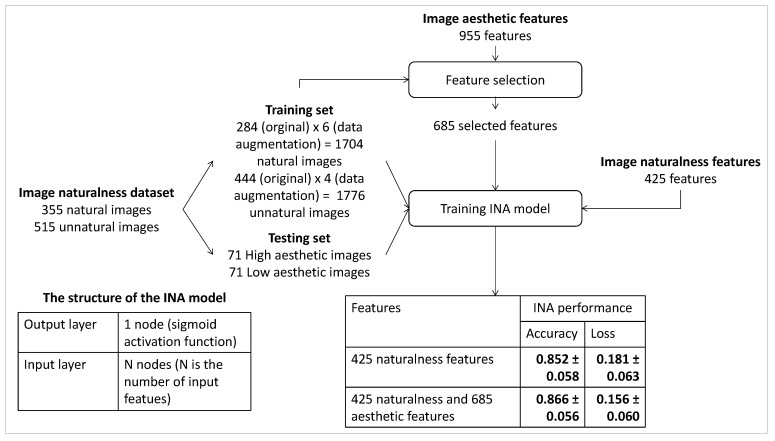
Experiment process of evaluating how IA features improves the performance of INA.

Similarly, INA based on IA features only is investigated in which the IA features include the 228 highly correlated the 669 moderately correlated, and the 127 weakly correlated features. The performance of INA based on IA features is compared with the one based on IN features only and the one based on the combination of IA features and IN features. The process of the experiment and the experiment results are presented in Figure 7. Starting with the considered IN dataset [1], in order to balance the number of natural and unnatural photos in the training set, a data augmentation process is applied to generate augmented versions of natural and unnatural photos by re-scaling, cropping and padding. Naturalness labels of augmented versions are kept the same as the labels of the original images. A training set (generated from 84% of the dataset) containing 1704 natural photos (284 original photos × 6 data augmented versions) and 1776 unnatural photos (444 original photos × 4 data augmented versions) and a testing set containing 71 natural photos and 71 unnatural photos (16% of the dataset without data augmentation) are extracted. The structure of the INA model and training parameters are set the same as in the previous experiments.

The experiment results in Figure 7 show that although the performance of the INA based on IA features is lower than the one based on IN features (0.835 ± 0.061 versus 0.852 ± 0.058), the result is quite good. The highly correlated and moderately correlated IA features could be the reason for the good performance of the INA based on IA features since the INA based on them has good results (0.814 ± 0.064 and 0.822 ± 0.063, respectively). Moreover, the results with the weakly correlated IA features are not good since the INA based on them has a much lower performance at 0.773 ± 0.069. The experiment’s results reflect that IA features do not help improve the INA performance significantly since the accuracy of INA based on IN features only is 0.852 ± 0.058, while this value of INA is based on the combination of IN and IA features increases insignificantly to 0.880 ± 0.053.

The obtained results prove that there is an overlap between IA and IN features and explain why the performance of IAA based on IN features only and the performance of INA based on IA features only are quite good. However, there are uncorrelated parts between IA and IN features. The performance of IAA and INA based on the uncorrelated features is not really good, so those IN and IA features might not be related to IAA and INA tasks, respectively. As a consequence, the combination of correlated and uncorrelated features does not help significantly improve the performance of IAA and INA.

### 3.3. Relationship between Naturalness/Unnaturalness and Low/High Aesthetics

#### 3.3.1. Are Natural Images High Aesthetic Ones and Unnatural Images Low Aesthetic Ones?

**Figure 8:**
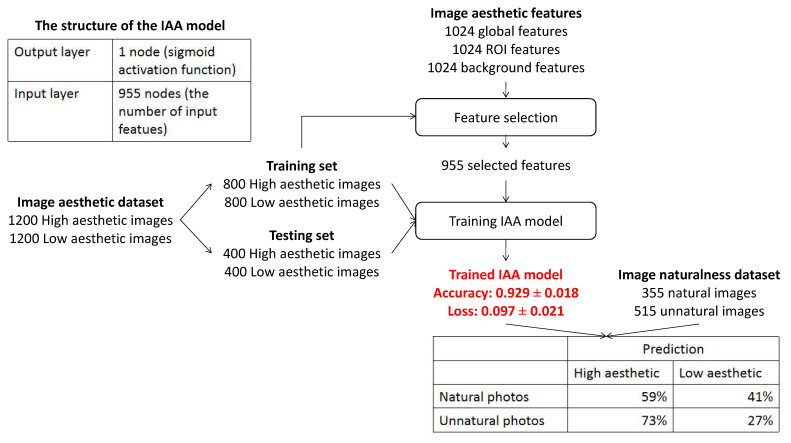
Process of the experiment evaluating the IA of the IN dataset images.

To answer this question, the IA of natural and unnatural images is investigated. Figure 8 shows the proposed process of evaluating the IA of the two image categories. In Section 3.2.1, the model based on the combination of IA features and IN features has the highest performance (0.946±0.016), so it is used to make the distinction between high aesthetic images and low aesthetic images. As a consequence, this model is used to assess the IA of natural and unnatural photos of the IN dataset [1].

According to the experiment’s results, 28% of the natural images are predicted as high aesthetic, and 61% of the unnatural images are predicted as low aesthetic. On the contrary, a significant part of natural photos (72%) is assessed as low aesthetic, and an insignificant part of unnatural photos (39%) is predicted as high aesthetic. Therefore, there is clearly not a cause to effect relation between naturalness and high aesthetics and unnaturalness and low aesthetics. Natural photos are classified as low aesthetic more often than unnatural ones. The reason could be the lack of post-processing in natural photos making those photos boring.

#### 3.3.2. Are High Aesthetic Images Natural Ones and Low Aesthetic Images Unnatural Ones?

**Figure 9:**
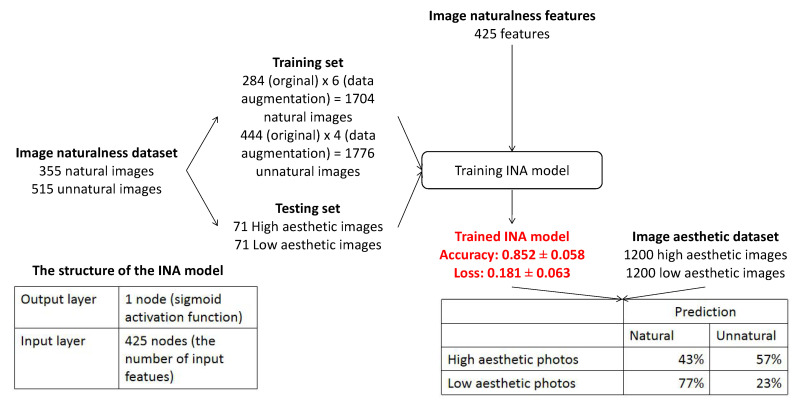
Process of the experiment evaluating the IN of photos in the IA dataset.

Similarly, in order to answer the question of this part, the IN of high and low aesthetic photos is investigated. The model that learned to assess IN is presented in Figure 9. According to Section 3.2.2, the INA model based on the combination of IA features and IN features has the best performance (0.880 ± 0.053), so it is used to classify natural and unnatural photos in this part. The IN of high aesthetic photos and low aesthetic photos of the IA dataset [35] is predicted by the INA model.

According to the experiment results, 35% of the high aesthetic photos are predicted as unnatural, while the majority of low aesthetic photos (89%) that are mostly not post-processed are assessed as natural. It appears that a high aesthetic photo does not mean a natural photo, and a low aesthetic photo is not always unnatural.

#### 3.3.3. IA and IN Score Correlation

Additionally, the IA and IN scores predicted by the IAA and the INA models on photos are considered as two vectors. The Pearson correlation and the Spearman rank correlation between the two vectors are computed, and the results are presented in Table 1. It appears that there is a weak negative correlation between the IA scores and the IN scores even on natural images, unnatural images, high aesthetic images, low aesthetic images or all images.

**Figure 10:**
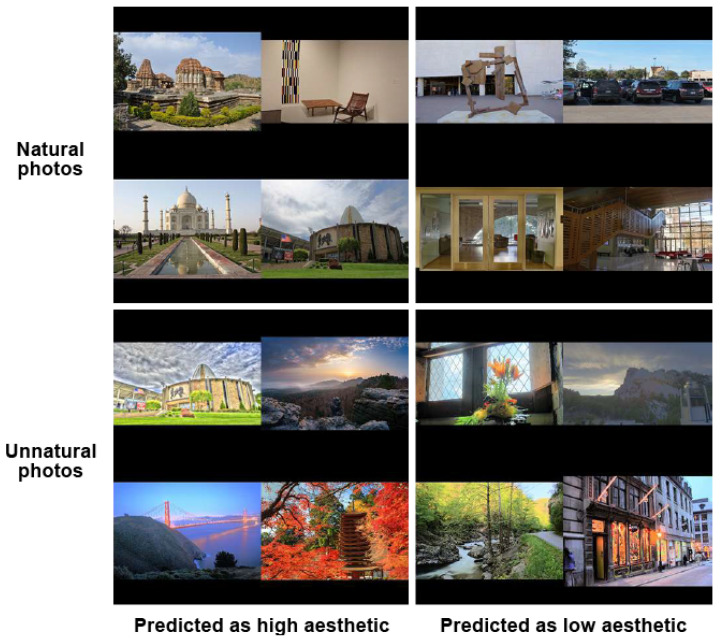
IAA samples of natural and unnatural images.

**Figure 11:**
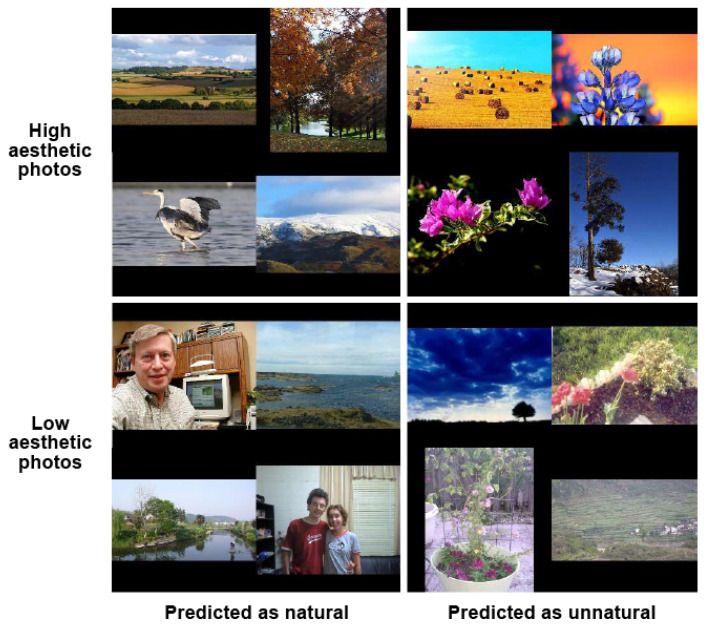
INA samples of high and low aesthetic photos.

The experiment’s results prove that there is no direct correlation between IA and IN since natural/unnatural photos are not always considered as high/low aesthetic, respectively, and vice versa. Samples of IAA and INA are presented in Figure 10 and Figure 11, respectively. Obviously, abusing enhancement methods that increase perceived aesthetic quality could provoke artifacts from imperceptible to obvious (over-enhancement), so the increase in IA could lead to a decrease in IN (see unnatural photos predicted as high aesthetic in Figure 10 and high aesthetic photos predicted as unnatural in Figure 11) or even to the decrease in IQ generally. On the contrary, when comparing a photo reproduced by an adjustment method, such as a tone-mapping operator and other single exposure versions, a tone-mapped photo could be more natural than a single exposure version of the same scene with deep, lively and realistic colors and contrast (see natural photos predicted as high aesthetic in Figure 10). Adjusted photos could be more appealing and interesting because of their uniqueness (compared with normal single exposure images that cannot preserve the high contrast and deep colors of the real scenes). However, when a photo is too faithful and familiar to observers, they might not be interested in the photo (see low aesthetic photos predicted as natural in Figure 11).

Generally, although IN and IA might have some correlations, they are nevertheless two different notions referring to two different aspects of IQ.

## 4. How Do IA and IN Affect Viewers’ IQ Perception in Different Contexts?

IQ, as well as IA and IN, are related to the quality of service [49] and quality of experience [50,51]. The quality of experience is based on human factors (individual properties, attitudes: visual and auditory acuity, gender, age, cognitive processes, socio-cultural and economic background, expectations) and contextual factors (experiment conditions: lightness, reference, distance, time, location). In contrast, the quality of service refers to photos’ properties and characteristics. The main question to be investigated is, “is IQA related to quality of experience or quality of service?”. Some people advocate that there are clear features and properties of photos deciding IQ, while opponents suggest that IQ is driven by individual opinions, experiences and context. The answer could be somewhere in the middle since individual feelings and experiment contexts have great influences on IQ perception, while intrinsic properties of photos can also affect IQ.

The main goal of this section is to study the relationships between IQ and the quality of experience. Specifically, the influence of IA and IN on IQ in different contexts is investigated. The influence of IA and IN in two cases, FRIQA and NRIQA, is considered. In order to clarify the relationships between IA, IN and IQ and how the influence of IA and IN on IQ is affected by experimental contexts, the idea is to investigate the correlation between IA and IQ and the one between IN and IQ in two cases: with and without reference.

**Figure 12:**
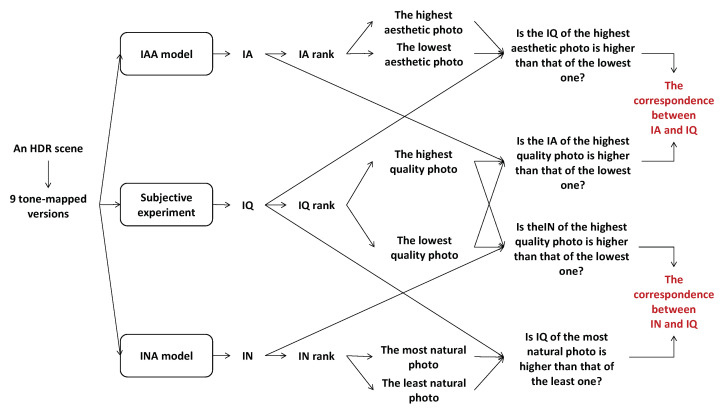
Process of estimating the correlation between IQ and IA and between IQ and IN.

**Figure 13:**
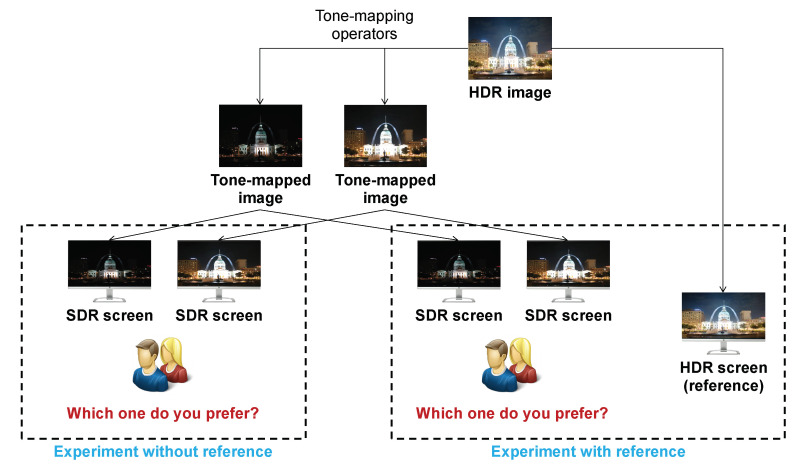
IQA experiment based on human preference with and without reference.

**Figure 14:**
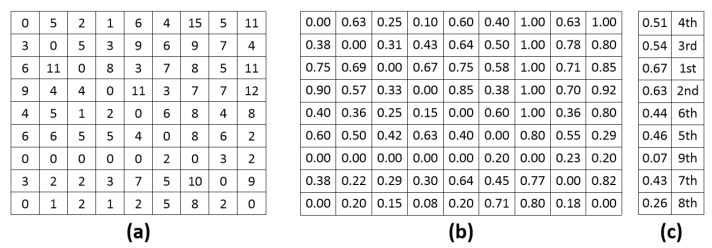
Process of pair comparison matrix analysis: (**a**) The pair comparison matrix of an HDR scene in which PCM[i,j] is the number of times $I_{i}$ is preferred when comparing $I_{i}$ to $I_{j}$, (**b**) Bradley–Terry score Matrix of each HDR scene, (**c**) IQ and ranks of tone-mapped versions of each HDR scene.

Figure 12 illustrates the whole process of estimating the correlation between IQ and IA and between IQ and IN. First, starting with the subjective IQA experiment presented in [10], 10 HDR scenes are considered, and 9 different tone-mapped versions are generated from each HDR scene by using simple linear clipping with inverse gamma correction, Drago [52], iCAM06 [53], Mantiuk [54] and Mai [55] tone-mapping operators with different parameter settings. These photos are categorized by scene, so there are nine tone-mapped versions and an HDR photo in each category. Possible tone-mapped image pairs in each category have been shown to 20 naive observers with and without reference. In their first test, observers had to choose which image they preferred between the two mapped versions of a given pair. Those images have been displayed on an SDR screen with a background luminance of 50 cd/m^2^ and a max luminance of 200 cd/m^2^. In the second test, not only a given pair but also a reference displayed on an HDR screen (background luminance of 100 cd/m^2^ and max luminance of 4000 cd/m^2^) was shown to viewers each time, and they had to answer the same question as in the first test (see Figure 13). The experiments were performed according to the ITU-R BT.500-11 for a subjective experiment. In those experiments, some images closer to the reference are preferred. However, some images less similar to the reference are sometimes preferred because they are more appealing. This subjective experiment is based on viewers’ global preference only: IA and IN are not mentioned in that experiment. Considering an HDR scene, Figure 14a shows the pair comparison matrix of the nine versions mapped from the HDR scene in which PCM[i,j] presents the number of times the *i*th tone-mapped version is preferred when comparing it to the *j*th version. It appears that the total number of observers for each pair is not the same, so the Bradley–Terry score Matrix is computed as in (3). The IQ of the *i*th tone-mapped version is then computed as in (4). The nine tone-mapped versions of each HDR scene are ranked based on IQ values. Figure 14b,c shows the Bradley–Terry score Matrix, IQ values and the IQ rank of the considered photos.

(3) $$BTM\left[i,j\right]=\frac{PCM[i,j]}{PCM[i,j]+PCM[j,i]}$$

(4) $$IQ\left[i\right]=\frac{1}{9}\sum_{j=1}^{9}BTM\left[i,j\right]$$

There are no subjective results of IA and IN for those tone-mapped images. Therefore, in the second step (cf. first line and third line of Figure 12), the IAA and INA models used in Section 3.3 are applied to the tone-mapped versions of each HDR scene in order to predict their IA and IN scores, respectively. First, the perception of IA is general; viewers focus on aesthetic criteria (how does the image satisfy their expectations) instead of technical criteria (how is the image generated). In addition to this, The IAA model was trained on a general image set containing different types of images (single exposure images, post-processed images, tone-mapped images), so it refers to general IA. Secondly, the INA model was trained on a dataset containing different types of images in which over 50% of the dataset are tone-mapped images, so it is able to predict the IN of tone-mapped images. That is the reason why the IAA model has been used on tone-mapped images in this case to evaluate the impact of IA and IN on IQ perception.

Output scores range from 0 to 1, referring to IA ([0, 0.5) means low aesthetic while [0.5, 1] means high aesthetic) and IN ([0, 0.5) means unnatural and [0.5, 1] means natural). Based on IA and IN scores, the photos are ranked from the highest score to the lowest score. According to IQ rank, the highest and the lowest quality photos of the scene are determined. The correlation between IQ and IA is evaluated by considering two questions: “is the IA rank of the highest IQ photo higher than that of the lowest one?” and “is the IQ rank of the highest IA photo higher than that of the lowest one?”. In a similar way, the correlation between IQ and IN is estimated by answering two sub-questions: “is the IN rank of the highest IQ photo higher than that of the lowest one?” and “is the IQ rank of the highest IN photo higher than that of the lowest one?”. The correlations between IQ and IA and the ones between IQ and IN are evaluated for the 10 HDR scenes with and without reference, and the results are presented in Table 2, in which the correlation score is calculated as the number of correlated cases (the higher IQ rank, the higher IN rank or the higher IA rank). It appears that in the case of NRIQA (experiment without reference), the correlation between IQ and IA is much higher than that between IQ and IN (correlation score: 15 versus 8). In contrast, in the case of FRIQA (experiment with reference), the difference between the two correlations decreases. The score of the correlation between IQ and IN is 7, while the score of the one between IQ and IA is 10. It seems that the influence of IA and IN on IQ is not the same for both NRIQA and FRIQA cases. The quality of experience might be the main cause of the differences. Specifically, IQ perception is affected by the visualization of the reference—a contextual factor of quality of experience. Without reference, viewers’ preference is mainly based on aesthetic perception. In contrast, IN has more influence on IQ than IA in the case of FRIQA.

Table 2 presents the correlation based on the comparison of IQ, IA and IN ranks between the highest IQ, IA and IN photos (the first photo in the lists ordered by IQ, IA and IN) and the lowest IQ, IA and IN photos (the ninth photo in the ordered lists) of nine versions generated from each HDR scene. Additional comparisons between the second and third highest IQ, IA and IN photos (the second and third photos in the ordered lists) and the second and third lowest IQ, IA and IN photos (the seventh and eighth photos in the ordered lists) are made to validate the assumption about the influence of IN and IA on IQ perception with and without reference. Correlations between IQ and IA and the ones between IQ and IN are estimated based on those comparisons and the Pearson correlation between IQ, IA and IN ranks. The obtained results are presented in Table 3. The experiment’s results prove that the assumption is true: without reference, IQ is more similar to IA than to IN, while with reference, the roles of IN and IA in IQ perception are more balanced.

It could be explained by the fact that in the case of NRIQA, viewers’ decisions are generally made based on individual feelings and perceptions. In fact, viewers tend to pay more attention to high aesthetic photos than to low aesthetic photos. Moreover, the lack of reference makes it difficult for viewers to assess IN since they have to use feelings and memory to evaluate IN. Therefore, they might focus on IA—an easier aspect to assess IQ in the case of NRIQA (correlation score: 117 versus 82, and Pearson correlation: 0.180 versus −0.070 for the correlation between IQ and IA and between IQ and IN, respectively). In contrast, with reference, viewers share their attention on both IA criteria and the similarity between the compared versions and the reference. Viewers’ preference is not only affected by visual aesthetic perception referring to IA but also by technical factors (visible artifacts, obvious differences) referring to IN. It explains why the correlation between IQ and IN and between IQ and IA are more balanced in the case of FRIQA (correlation score: 94 vs. 90m and Pearson correlation: 0.032 vs. 0.028 for the correlation between IQ and IN and between IQ and IA, respectively).

## 5. Conclusions

There are two main contributions in this paper related to the correlations between IA and IN in relation to IQ. First, the relationships between IA and IN were investigated. The experiment’s results prove that the correlation of IA features to IN features is quite significant, but the correlation of IN features to IA features is much lower. Additionally, the obtained results reflect that a high aesthetic photo does not mean a natural photo and a natural photo is not always considered a high aesthetic photo. Further, IA and IN features do not help significantly improve the performances of INA and IAA, respectively. In conclusion, although there are few moderate correlations and overlaps between IA and IN, they are two different notions reflecting different aspects of IQ. Secondly, the influences of IA and IN on IQ were evaluated, and it appears that those influences are not the same depending on the experimental context (FRIQA or NRIQA). The main cause of the differences is the quality of experience since contextual factors change observers’ preferences. The experiment’s results refer to the fact that viewers’ IQ perception is more related to IA than to IN in NRIQA since NRIQA is mostly based on individual feelings and visual aesthetic perception. In contrast, the influence of IN and IA on IQ is more balanced in FRIQA because FRIQA is related to both individual opinions and technical aspects (technical errors, artifacts and specific screen), reflecting the similarity between transformed versions and original versions.

According to the current results, the direction of our future research is to develop algorithms able to enhance IQ based on both IA and IN aspects. Studying IA and IN and analyzing the correlations between IQ, IA and IN to understand the positive influences and negative effects on IQ could be considered the first step. The second step will be an improvement of IQA performance by considering IA and IN components. These two steps are the basis for developing methods to improve IQ by restoring the naturalness of detected unnatural images and enhancing the aesthetic quality of detected low-aesthetic images.

## Figures and Tables

**Table 1:** The correlation between IA scores and IN scores.

Correlation between IA and IN Scores Computed on	Pearson Correlation	Spearman Rank Correlation
Natural images	−0.078	−0.105
Unnatural images	−0.129	−0.066
High aesthetic images	−0.094	−0.145
Low aesthetic images	−0.139	−0.087
All images	−0.191	−0.218

**Table 2:** Correlation between IQ and IA and IN in the two cases FRIQA and NRIQA. IQ vs. IN: “Is the IN rank of the highest IQ photo higher than that of the lowest one?”. IN vs. IQ: “Is the IQ rank of the highest IN photo higher than that of the lowest one?”. IQ vs. IA: “Is the IA rank of the highest IQ photo higher than that of the lowest one?”. IA vs. IQ: “Is the IQ rank of the highest IA photo higher than that of the lowest one?”. Y: Yes. N: No. Score means correlation score.

			Scenes										
	Correlation		1	2	3	4	5	6	7	8	9	10	Score
NRIQA	between IQ and IN	IQ vs. IN	Y	N	Y	Y	N	N	Y	N	N	N	8
		IN vs. IQ	Y	N	Y	Y	N	N	Y	N	N	N	
	between IQ and IA	IQ vs. IA	Y	Y	Y	Y	N	Y	N	Y	Y	Y	15
		IA vs. IQ	N	N	Y	Y	Y	Y	Y	Y	N	Y	
FRIQA	between IQ and IN	IQ vs. IN	Y	N	Y	N	N	N	Y	N	N	N	7
		IN vs. IQ	Y	N	Y	N	N	Y	Y	N	N	N	
	between IQ and IA	IQ vs. IA	Y	Y	N	Y	Y	N	Y	N	Y	N	10
		IA vs. IQ	Y	Y	N	N	Y	N	N	Y	N	N	

**Table 3:** Correlations between IQ and IA and IN in the two cases FRIQA and NRIQA.

	**NRIQA**		**FRIQA**	
**Comparison** **between**	**Correlation score between**			
	**IQ and IN**	**IQ and IA**	**IQ and IN**	**IQ and IA**
1st vs. 9th	8	15	7	10
1st vs. 8th	9	10	9	9
1st vs. 7th	9	14	10	10
2nd vs. 9th	10	17	9	13
2nd vs. 8th	7	13	13	13
2nd vs. 7th	8	16	11	10
3rd vs. 9th	10	11	9	10
3rd vs. 8th	11	11	15	7
3rd vs. 7th	10	10	11	8
Total	82	117	94	90
	**Pearson correlation between**			
	**IQ and IN** **ranks**	**IQ and IA** **ranks**	**IQ and IN** **ranks**	**IQ and IA** **ranks**
	−0.070	0.180	0.032	0.028

## Data Availability

1. Dataset “Pair Comparison between TMO images”—https://ieeexplore.ieee.org/document/7148103. 2. Dataset “Image naturalness”—https://www.sciencedirect.com/science/article/abs/pii/S1077314220300485. 3. Dataset “CUHKPQ”—https://ieeexplore.ieee.org/document/6544270.

## Abbreviations

The following abbreviations are used in this manuscript:

CNN	Convolutional Neural Network
FRIQA	Full Reference Image Quality Assessment
IA	Image Aesthetic
IAA	Image Aesthetic Assessment
IN	Image Naturalness
INA	Image Naturalness Assessment
IQ	Image Quality
IQA	Image Quality Assessment
NRIQA	No Reference Image Quality Assessment
